# Supplementary material for: Myokine mediated muscle-kidney crosstalk suppresses metabolic reprogramming and fibrosis in damaged kidneys
Source: Nat Commun. 2017 Nov 14;8:1493. doi: 10.1038/s41467-017-01646-6 (PMC5686208; doi:10.1038/s41467-017-01646-6)
Supplement: Supplementary file 1 — Supplementary Information [file 41467_2017_1646_MOESM1_ESM.pdf]

Supplementary Figure 1

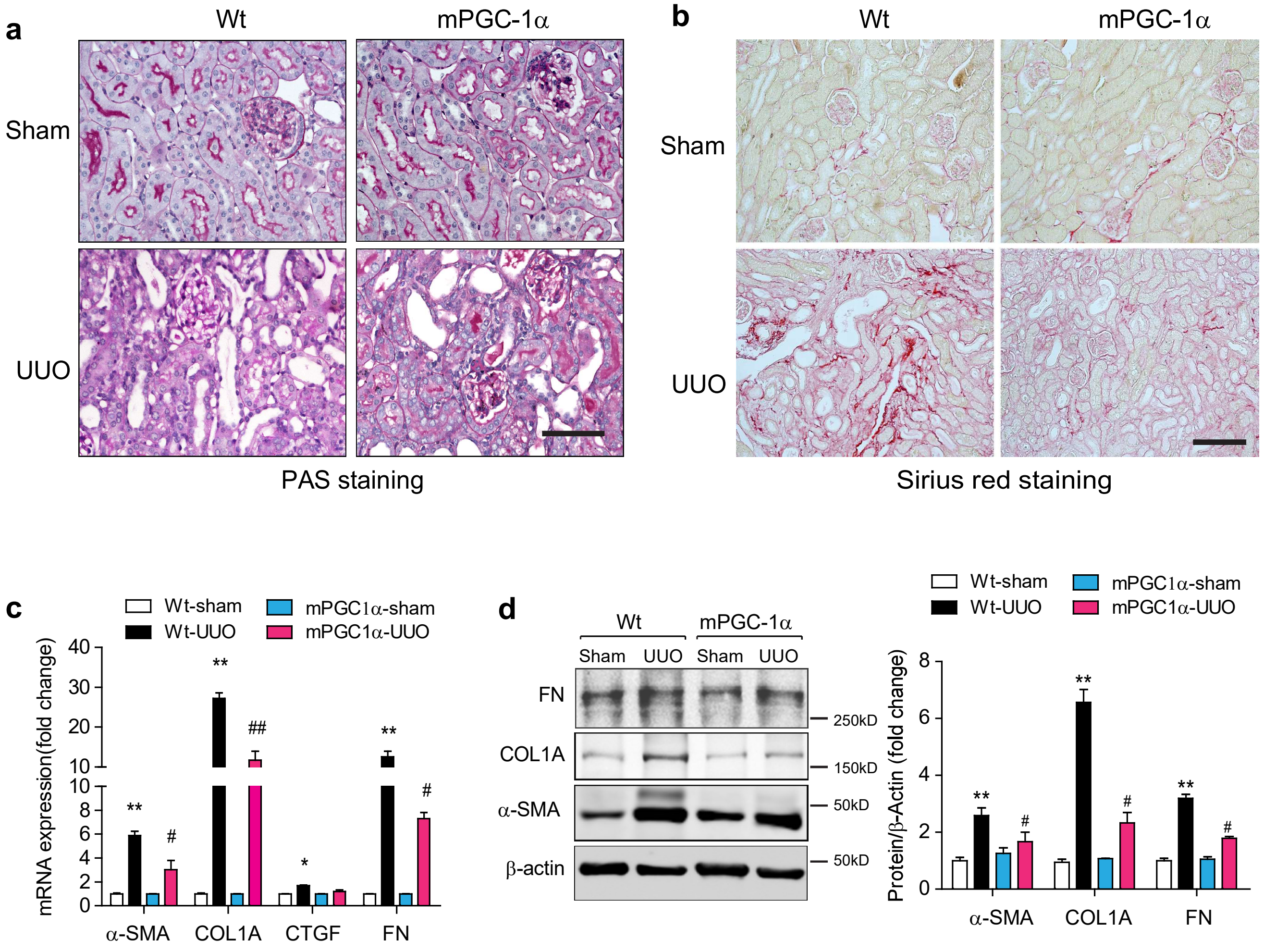

Supplementary Fig. 1: PGC-1α overexpression only in muscles limits renal fibrosis induced by UUO

a: Reduced tubule dilation, atrophy and brush-board loss in kidneys of mPGC-1α mice with UUO for 7 days. Scale bars, 50 µm.

b: Less collagen deposition in kidneys of mPGC-1α mice with UUO for 7 days.
Scale bars, 100 µm.

c: Real-time quantitative PCR measurements of α-SMA, Collagen 1A, Connective Tissue Growth Factor (CTGF) and Fibronectin (FN) mRNA expressions in kidneys of Wt and mPGC-1α mice with or without UUO.

d: Fibrotic proteins (α-SMA, COL1A expression and FN) expression in kidney from each group was assessed with western blotting. Statistical analysis (c, d) was presented as mean± s.e.m.; *$p<0.05$ or **$p < 0.01$ for Wt-UUO vs. Wt-Sham and #$P < 0.05$ or ##$p<0.01$ for mPGC-1α-UUO vs. Wt-UUO (n=5 per group, on-way ANOVA with Bonferroni's multiple comparison test.

Supplementary Figure 2

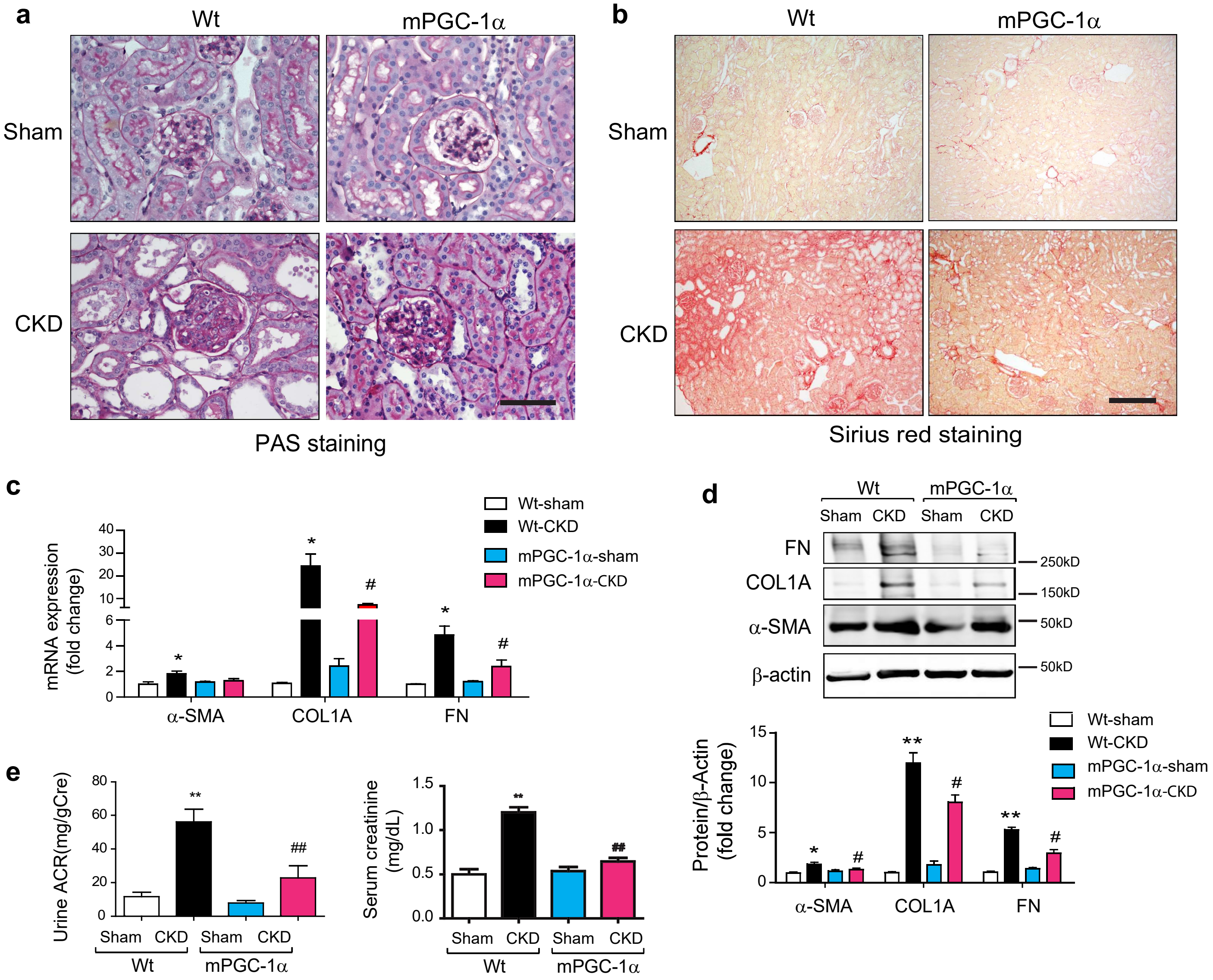

Supplementary Fig. 2: muscle-specific PGC-1α overexpression improved kidney function and suppressed fibrogenesis in subtotal nephrectomy kidney (CKD)

a: PAS staining shows glomerulosclerosis and tubule dilation in kidneys with subtotal nephrectomy for 3 months. Scale bars, 50 μm.

b: Reduced collagen deposition in kidneys of mPGC-1α mice with subtotal nephrectomy for 3 months. Scale bars, 100 μm.

c: mRNA expression of a-smooth muscle actin (α-SMA), Collagen 1A (COL1A) and Fibronectin (FN).

d: Western blot analysis of α-SMA, COL1A and FN in kidneys from each group as indicated.

e: Proteinuria (albumin-creatinine ratio, ACR) and serum creatinine in mice with subtotal nephrectomy.

All statistic results (c,d,e) were presented as mean± s.e.m.; *p<0.05 or **p<0.01 for Wt-CKD vs. Wt-Sham; # p<0.05 or ##p<0.01 for mPGC-1α-CKD vs. Wt-CKD (n=6 per group, on-way ANOVA with Bonferroni's multiple comparison test).

Supplementary Figure 3

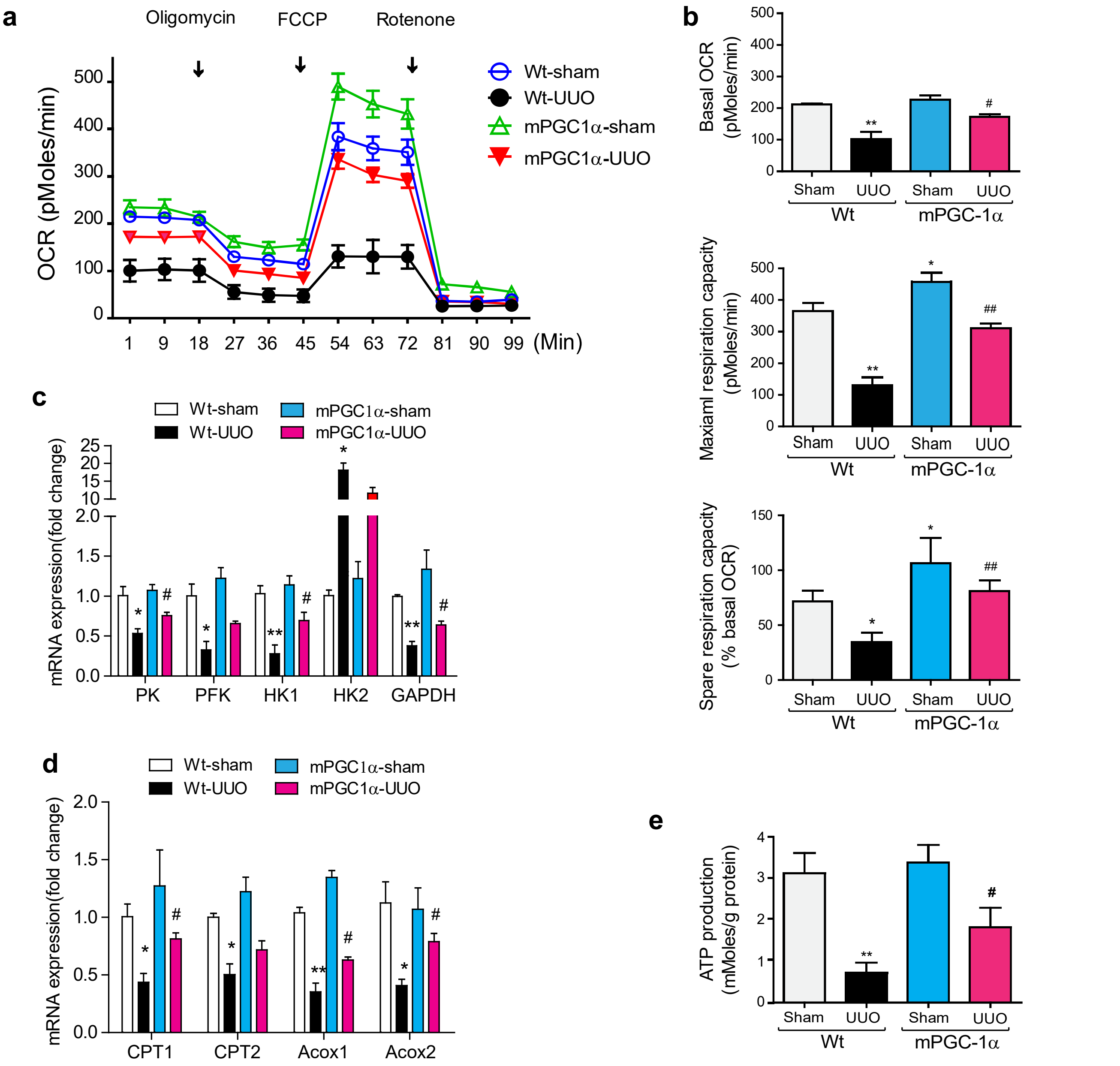

Supplementary Fig. 3: muscle-specific PGC-1α overexpression improved energy metabolism in kidney tubular cells from mice with 7-day UUO

a: Cellular respiration in fresh isolated tubules was assessed with Oxygen Consumption Rate (OCR) and Extracellular Acidification Rate (ECAR) with mitochondrial respiration inhibitors.

b: Measurements of basal, maximal respiration and ATP-linked respiration in tubules from each group.

c: mRNA expression of enzymes in glucose metabolism (Real-time qPCR).

d: mRNA expression of enzymes in fatty acid oxidation pathway (Real-time qPCR).

e: ATP concentration in kidney cortex from each group as indicated. All statistical data (b, c, d and e) were presented as mean± s.e.m.; *p<0.05 or **p<0.01 for Wt-UUO vs. Wt-Sham; # p<0.05 or ##p<0.01 for mPGC-1α-UUO vs. WT+UUO (n=5 per group, on-way ANOVA with Bonferroni's multiple comparison test).

Supplementary Figure 4

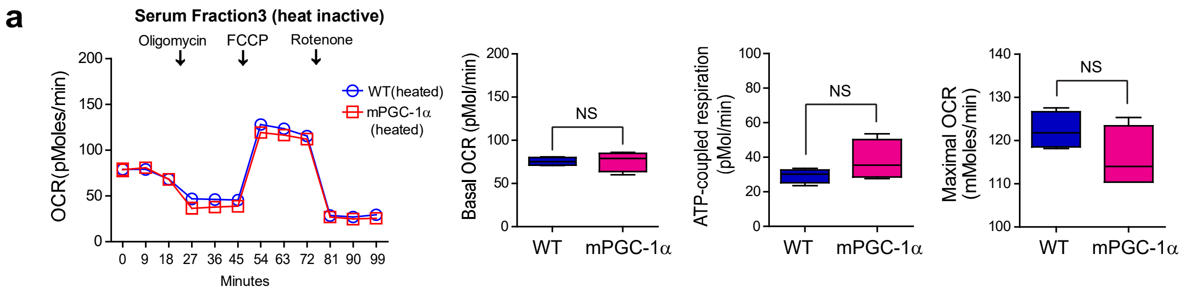

Supplementary Fig. 4: Myokines in serum from mPGC-1α mice stimulate tubular cell respiration
a: Serum fraction 3 (<50kd, but >10kd) from Wt or mPGC-1α mice was boiled for 10 min. Primary tubule cell cultures were then treated with boiled sera for 12 h following an assessment of cellular respiration. Serum effects on cell respiration was eliminated after heat inactivation. Data was presented as mean± s.e.m.; NS indicates nonsignificant (t-tests; n=3 per group).

Supplementary Figure 5

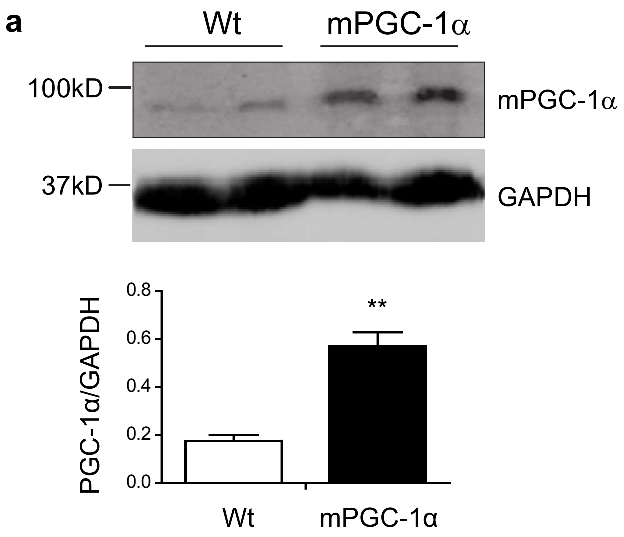

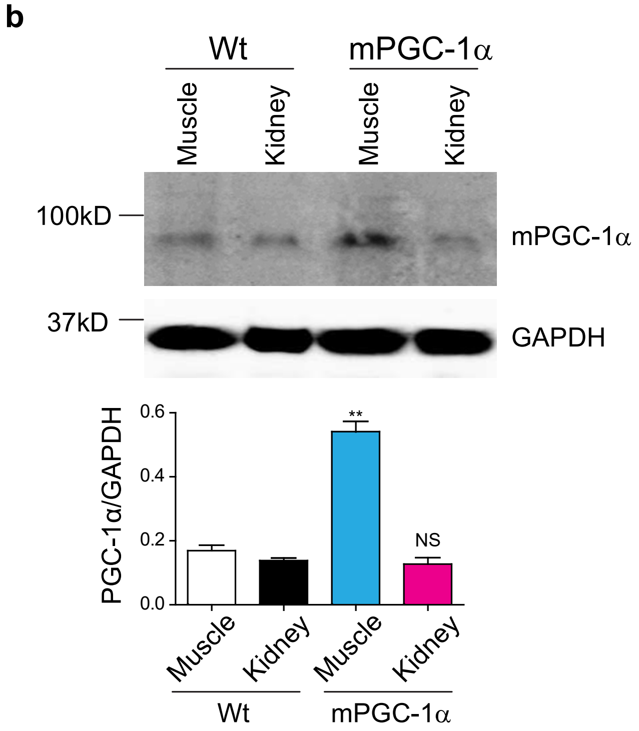

Supplementary Fig. 5: PGC-1α expression in muscle and kidney from wild type or mPGC-1α mice
a: To achieve reliable results, we crossed muscle-specific PGC-1α transgenic C57BL/6 mice with CD1 strain breeders because, CD1 mice have less resistance to kidney injury. After backcrossing with CD1 mouse for 3 generations, overexpression of muscle-specific PGC-1 transgene was confirmed in TA muscles from mPGC-1α mice. Western bolt revealed a 3-fold increase in PGC-1α in muscles of mPGC-1α mice vs. the values in muscles of wildtype mice.
Data was presented as mean± s.e.m.; **p<0.01(n=3 per group, Student t-test).
b: To determine if there is possible mPGC-1α transgene leakiness in kidney, we compared PGC-1α protein expression in muscle and kidney from wild type or mPGC-1α mice. Western blot analysis showed that PGC-1α highly expressed in muscle of mPGC-1α mice, but this induction of muscle PGC-1α was not companied with increase in PGC-1α in kidney. Data was presented as mean± s.e.m.; **p<0.01(n=3 per group, Student-Newman-Kuel's two-tailed, unpaired test).

Supplementary Figure 6

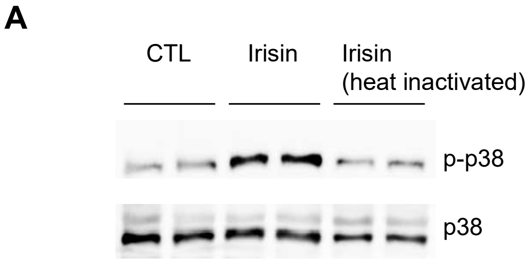

Supplementary Fig. 6: Heat-inactivated irisin did not stimulate p38 phosphorylation
A: primary kidney tubule cell cultures were treated with PBS, irisin (2ug/lm) or irisin but boiled for 10 min before treatment. After incubation at 37C for 1h, cells were lysis in RIPA buffer and the cell lysate was subjected to western blotting using anti p-p38 antibody, the non-phosphorylated p38 was used as loading control. Because endotoxin is heat resistant, the result indicated that the level of endotoxin is very low in the recombinant irisin.

Supplementary Figure 7 The sequences of primers used in this study:

Genes	Forward	Reverse
ANGPTL4	CCTGTGGTAACGCTTGTCAG	TGCTGGATCTTGCTGTTTTG
BDNF	GCCCAACGAAGAAAACCATA	GCTGTGACCCACTCGCTAAT
Cathepsin L1	TTAGTGCAGAGTGGCACCAG	GTTGCTGTATTCCCCGTTGT
CTSB	TCCTTCTTTCTTGCCTGCTG	GTGCCACACAGCTTCTTCAG
CTSD	CAACAGAAGCTGGTGGACAA	TAGGCCTTTCGAGTGACGTT
Decorin	TCTATGTGCCCCTACCGATG	GAAGGCCCCTTCTTTGATCT
FGF-2	AGCGGCTCTACTGCAAGAAC	GCCGTCCATCTTCCTTCATA
FGF-21	CTGGGGGTCTACCAAGCATA	CACCCAGGATTTGAATGACC
Follistatin-like1	CAATGTGCCGTCACAGAGAA	GGATCTTGGATCCAGTGAGG
IGF-1	GAAAATCAGCAGCCTTCCAA	GTCTCTGGTCCAGCTGTTGGT
IL-6	CCGGAGAGGAGACTTCACAG	TCTGCAAGTGCATCATCGTT
IL-7	GGGGTCCTGGGAGTGATTAT	GTGACAGGCAGCAGAACAAG
IL-10	TGCTATGCTGCCTGCTCTTA	ATGTTGTCCAGCTGGTCCTT
IL-15	TGAGGAATACATCCATCTCGTG	TGGCCTCTGTTTTAGGGAGA
Irisin	GCTAGGCTGCGTCTGCTTC	AGCCAATGACCACTTCATCC
LIF	CGCCAATGCTCTCTTCATTT	ACCATCCGATACAGCTCCAC
LUM	TGCTCGAGCTTGATCTCTCC	AAGCGCAGATGCTTGATCTT
MCP-1	CAGGTCCCTGTCATGCTTCT	TCATTGGGATCATCTTGCTG
MSTN	ACGCTACCACGGAAACAATC	GGAGTCTTGACGGGTCTGAG
Myonectin	CCCCTTTATCCCATCTGAGG	AGTGAGATCCCTGGTGCAGT
PAI-1	TTCTCTCCCTATGGCGTGTC	ATGAGCTCCTTGGAGAGCTG
PEDF	CCTGTGTGCTACTCCTCTGGA	GACCTTGAAGAAGGGGTCCT
SPARC	AACCCAGTCCAGGTGGAAAT	CTCTCGTCCAGCTCACACAC
VEGF	CAGGCTGCTGTAACGATGAA	GCATTCACATCTGCTGTGCT
GAPDH	TGTGATGGGTGTGAACCACGAGAA	CATGAGCCCTTCCACAATGCCAAA
CPT1	GCTCTACATCACCCCAACCC	GCAGAGCAGAGGGGAATTGT
CPT2	GTATCTGCAGCACAGCATCG	CTGGCTGTCATTCAAGAGAGG
ACOX1	GCGTTACGAGGTGGCTGTTA	AGGAACATGCCCAAGTGAAG
ACOX2	CCAGCACTTTGAGGAGGAGA	GGACTTGGCTTCCTTTAGGG
Hk-1	CTCACCAGGGCTACTGAGGA	CCGCATGGCATACAGATACTT
Hk-2	ATGATCGCCTCGCATATGAT	TAGCCCCTTCTCCATCTCCT
Pk(m)	GACGTCCGCTCTAGGTATCG	TGTGTTCCAGGAAGGTGTCA
Pk(LR)	CAGGTACGCAGCAGTATGGA	TCCAGAAGGCAGAGGTGTTC
Pfk(L)	TACCGTGGACCTGGAGAAAC	AGACTTTGGCCCCCACATA
Pfk(P)	CGAAGTACCTGGAGCACCTC	CCCGTGTAGATTCCCATGC
RPL39	TTTCCGAATCAAGCGATTCCTGGC	AGCTTCGTTCTCCTCCAGTGTCTT

Supplementary Figure 8

Orginal western blots for Figure 1

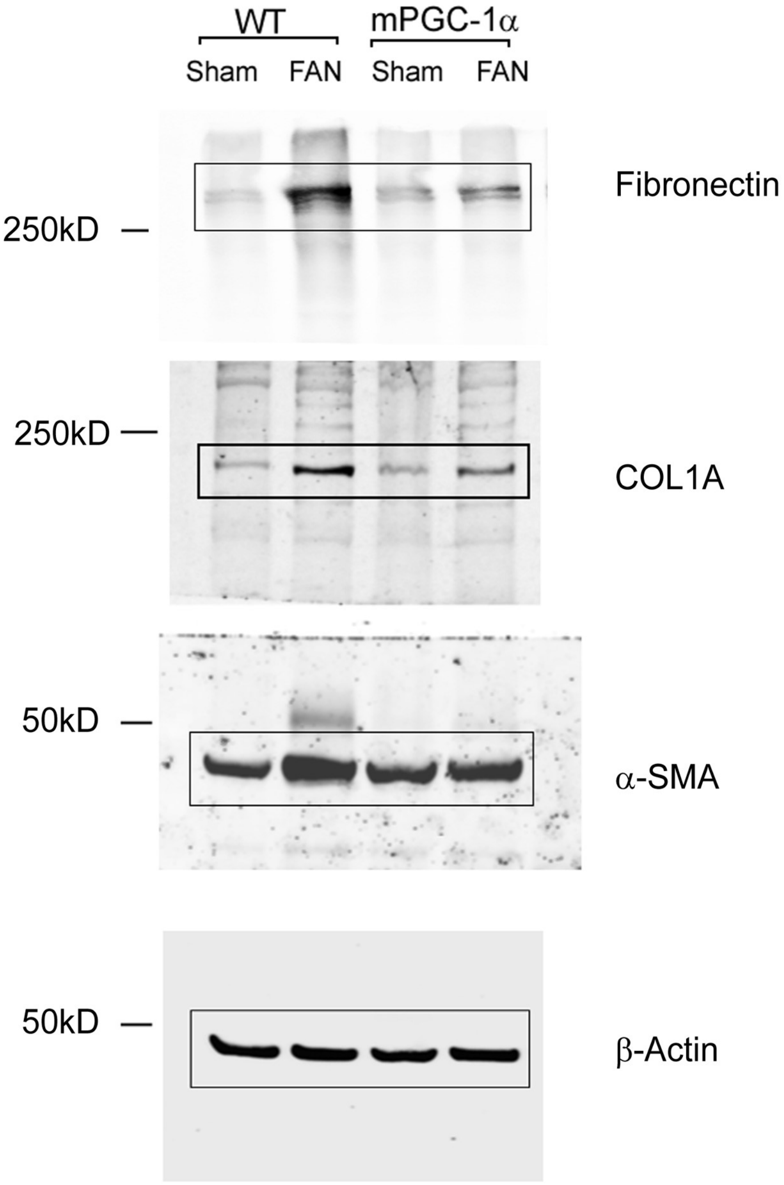

Orginal western blots for Figure 2

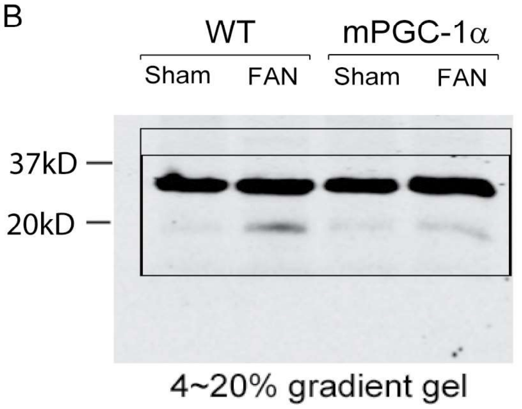

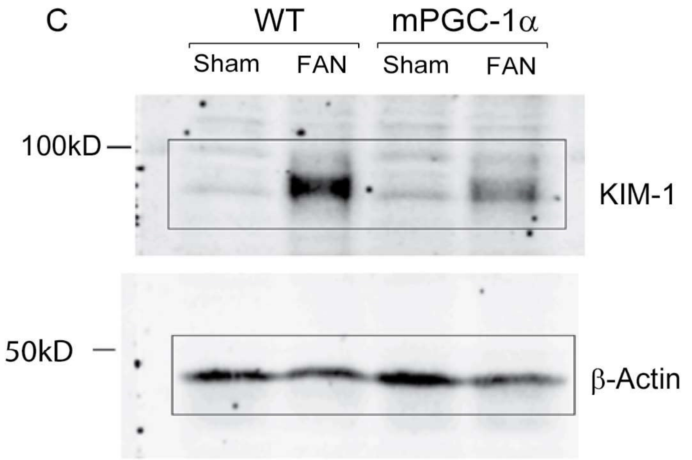

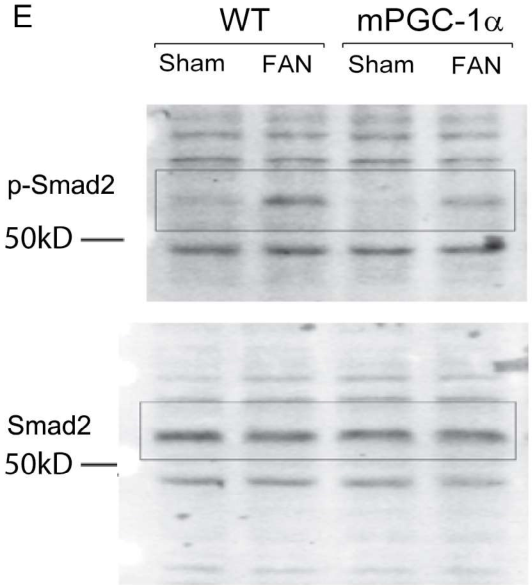

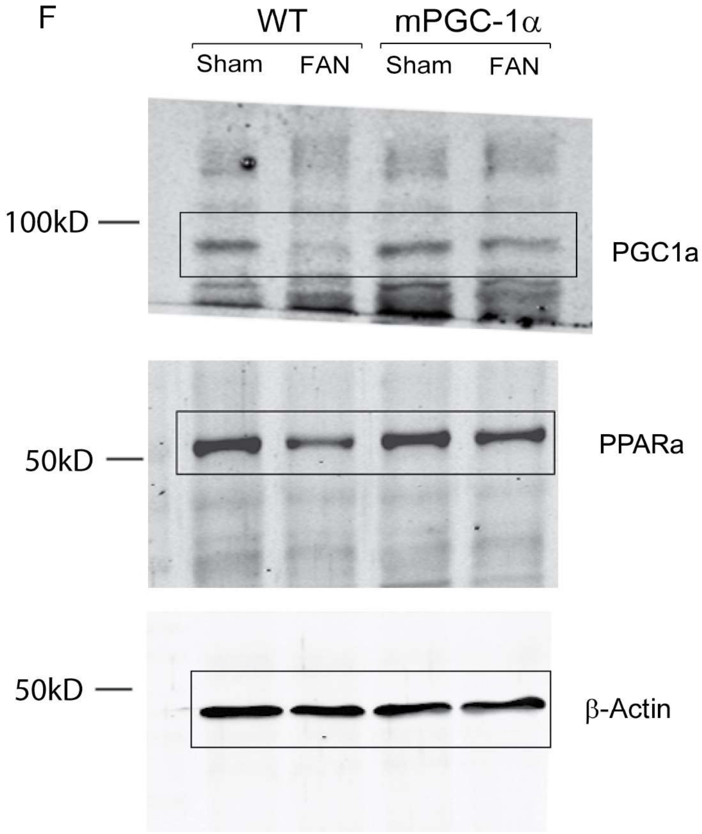

Orginal western blots for Figure 5

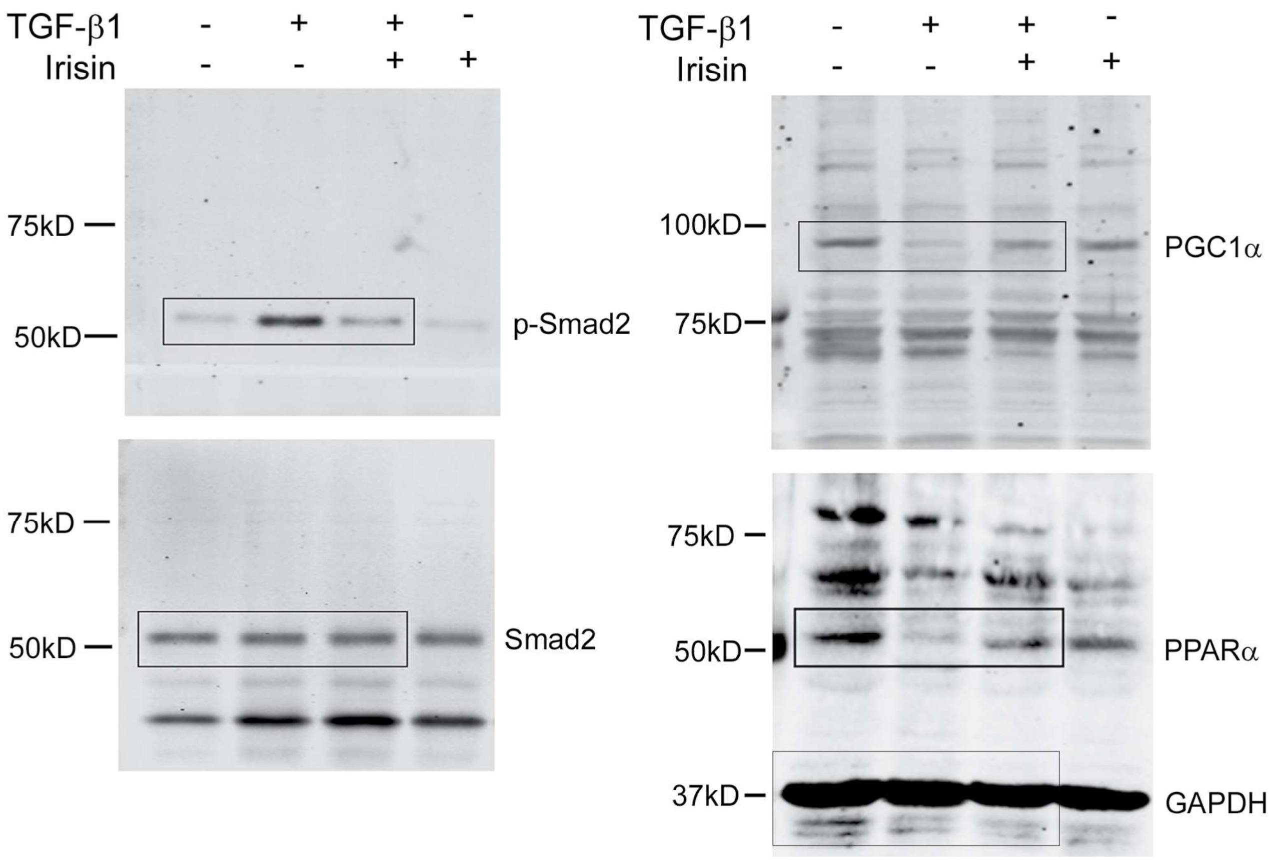

Orginal western blots for Figure 6

Fig 6 C

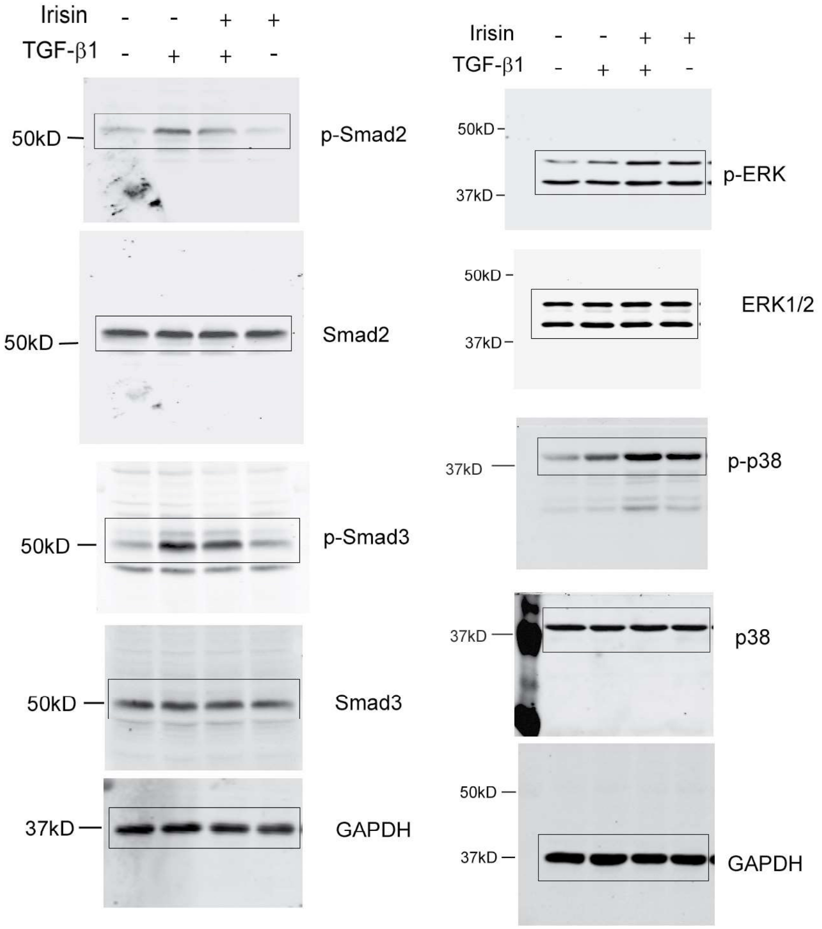

Fig 6D

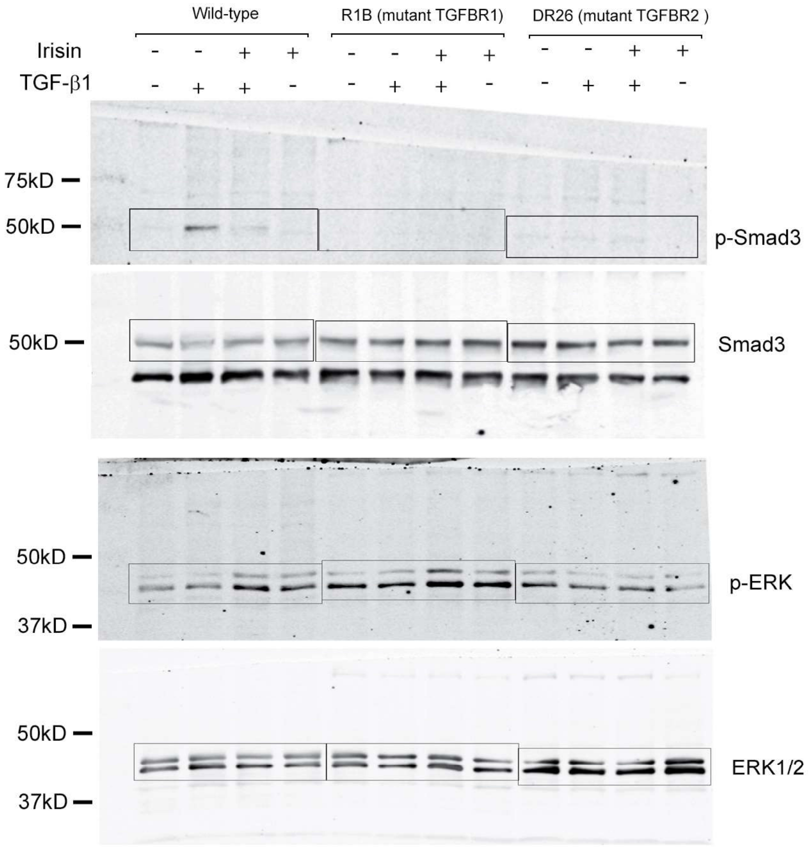

Orginal western blots for Figure 6

Fig 6 E

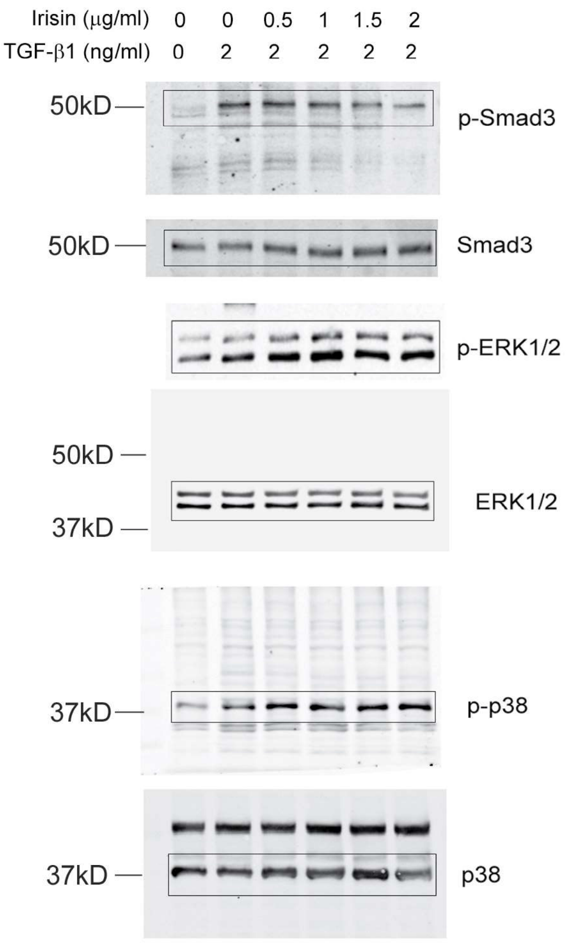

Fig6 F

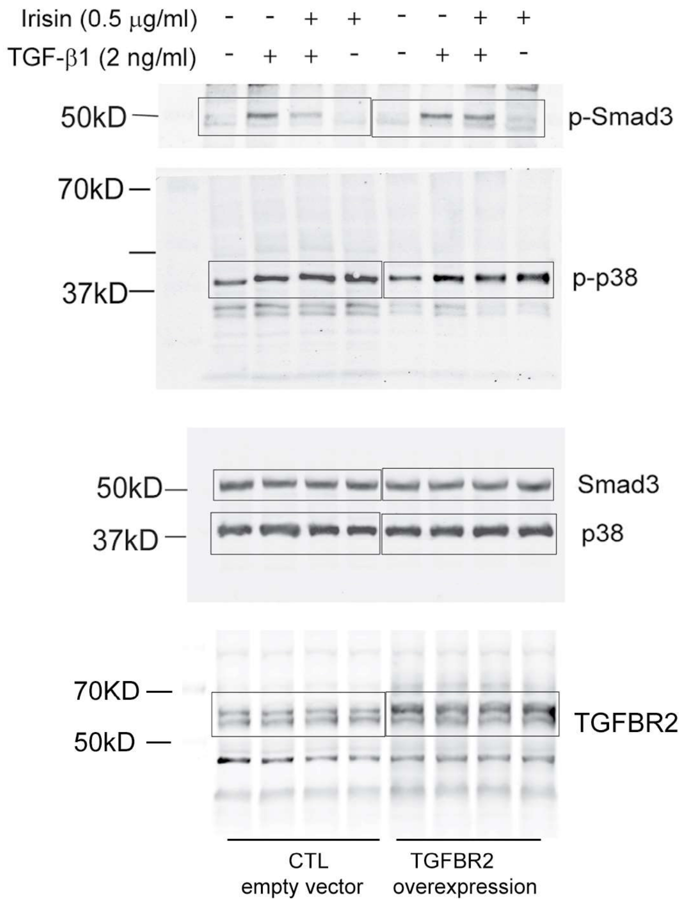

Orginal western blots for Figure 7

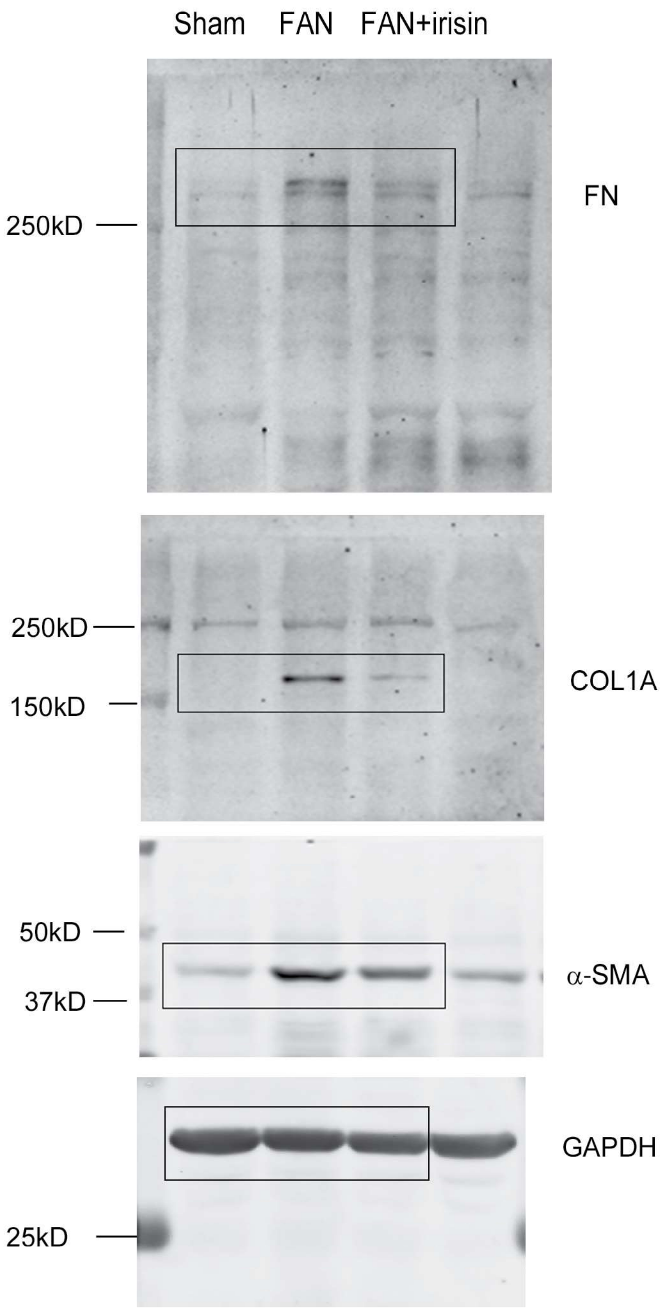

Orginal western blots for Supplementary Fig. 1

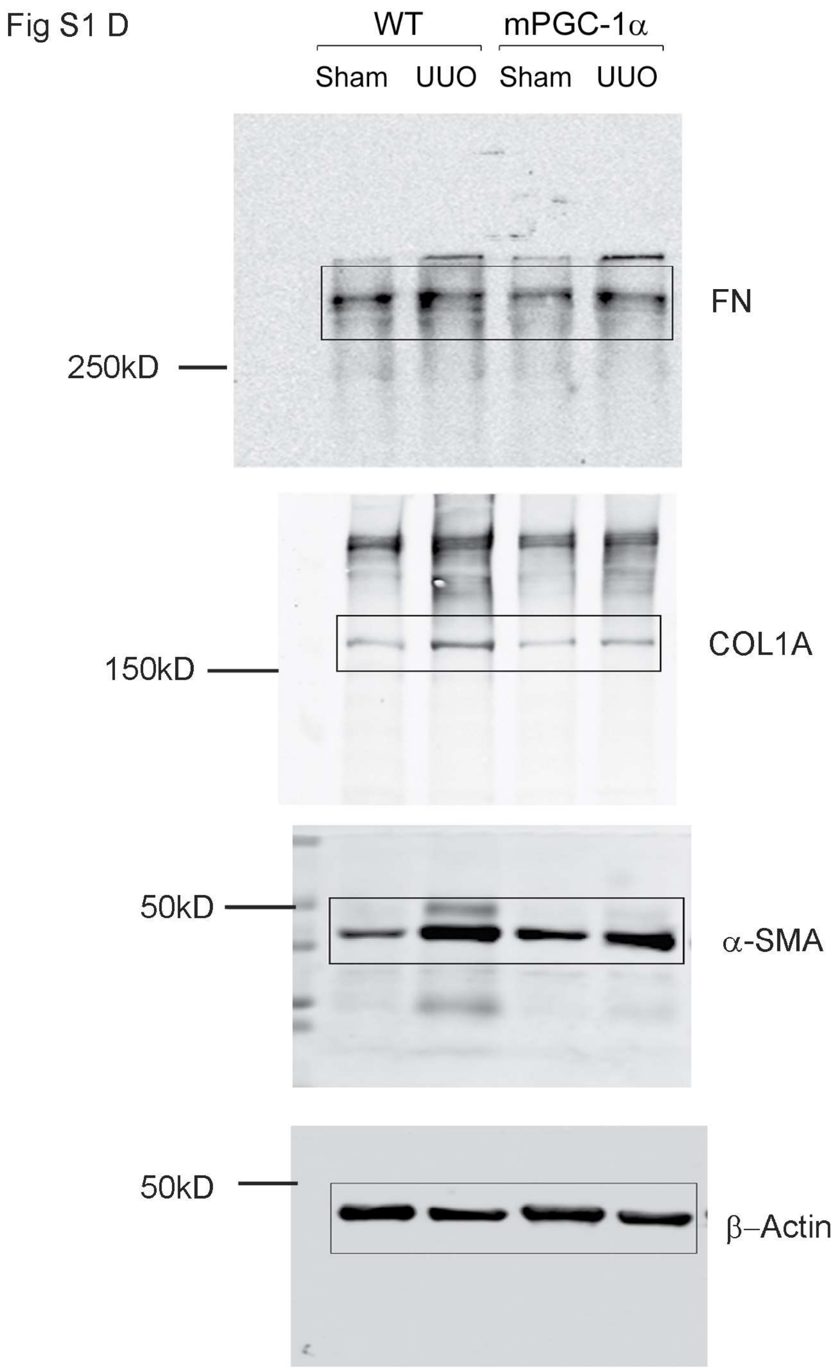

Orginal western blots for Supplementary Fig. 2

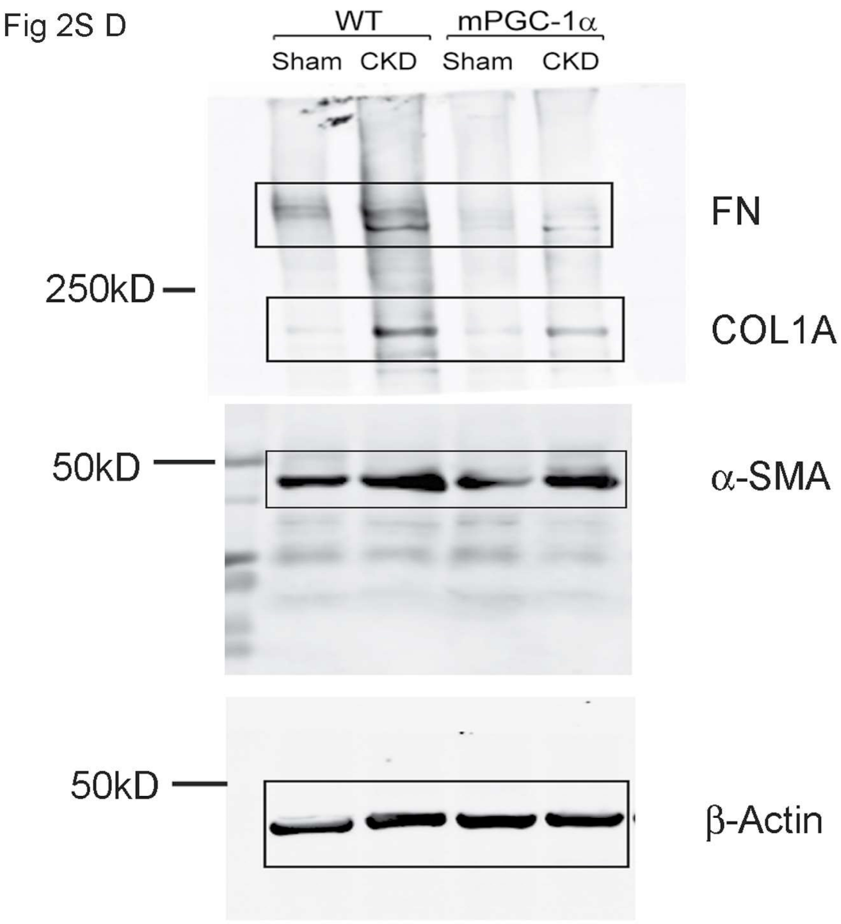

Supplementary table 1 **Phospho-Protein antibody array (TGF-β1 signaling pathway)**

Name	ratio of PP/TP over ctl		
	tgfb1	tgfb1-irisin	irisin
Abl1 (Phospho-Thr754/735)	0.377548032	0.74069257	0.426950755
Abl1 (Phospho-Tyr204)	0.412376015	0.676827524	0.480374104
Abl1 (Phospho-Tyr412)	0.454929567	0.778227276	0.589848092
AKT (Phospho-Ser473)	1.330623246	0.930540052	1.363322485
AKT (Phospho-Thr308)	1.224383464	1.059432374	1.243796367
AKT (Phospho-Tyr326)	0.891410061	0.628487788	0.980669162
AKT1 (Phospho-Ser124)	1.545171137	0.828575653	1.623872914
AKT1 (Phospho-Ser246)	1.455177045	0.847340971	1.460732875
AKT1 (Phospho-Thr450)	1.429749534	0.968111916	1.558176393
AKT1 (Phospho-Thr72)	1.345324448	1.010148261	1.251136571
AKT1 (Phospho-Tyr474)	1.369769921	1.051505065	1.281564128
AKT2 (Phospho-Ser474)	1.171913877	1.017126946	1.311727979
c-Abl (Phospho-Tyr245)	1.249691078	0.919007122	1.060478096
c-Abl (Phospho-Tyr412)	1.280890825	1.04355015	1.145308532
CBP (Inter)	3.636011266	1.113448892	2.355008318
cofilin (Phospho-Ser3)	1.270161473	1.41235456	1.253354977
ERK1-p44/42 MAP Kinase (Phospho-Thr202)	1.241408094	1.234269253	1.467629818
ERK1-p44/42 MAP Kinase (Phospho-Tyr204)	1.255672168	1.198962098	1.407232037
ERK3 (Phospho-Ser189)	0.60561755	0.779698475	0.786850451
ERK8 (Phospho-Thr175/Tyr177)	1.294273982	1.037615625	1.513742379
Gab2 (Phospho-Tyr643)	0.727439463	2.036730103	0.710495207
JNK1/2/3 (Phospho-Thr183/Tyr185)	1.070242145	1.577572677	1.368961572
JNKK (MKK4) (Inter)	4.70847976	1.050823031	3.811622255
LIMK1 (Phospho-Thr508)	1.233724636	1.104005249	0.991237636
MAP3K1/MEKK1 (Phospho-Thr1381)	5.678512386	1.40987172	5.383408722
MKK3 (Phospho-Ser189)	1.113530414	0.71954932	1.165032515
MKK3/MAP2K3 (Phospho-Thr222)	0.967971017	1.143325849	0.869931483
MKK6 (Phospho-Ser207)	1.238804697	1.104990249	1.540089733
mTOR (Phospho-Ser2448)	1.346436391	1.354957468	1.603687542
mTOR (Phospho-Ser2481)	1.071607781	1.071458677	1.250555131
mTOR (Phospho-Thr2446)	1.044751394	1.427777001	1.14798249
Myc (Phospho-Ser373)	1.169204221	0.71040377	1.006093669
Myc (Phospho-Ser62)	1.192097107	0.624797981	1.028289054
Myc (Phospho-Thr358)	1.445713374	0.820624813	1.288264308
Myc (Phospho-Thr58)	1.137333412	0.705393542	0.977297339
p300/CBP (C-term)	5.143474235	1.024676268	5.288818792
p38 MAPK (Phospho-Thr180)	1.040679457	0.959009629	0.883945406
p38 MAPK (Phospho-Tyr182)	1.074804024	1.033958856	0.898629735
p38 MAPK (Phospho-Tyr322)	0.964519881	0.844921752	1.015053671
PAK1 (Phospho-Ser204)	0.93085273	1.279047116	0.923696044
PAK1 (Phospho-Thr212)	1.116559163	1.193476698	1.171695241
PAK1/2 (Phospho-Ser199)	1.490830544	1.040705196	2.186139045
PAK1/2/3 (Phospho-Ser141)	0.840677434	0.770427276	1.084191685
PAK1/2/3 (Phospho-Thr423/402/421)	0.724592251	0.90397559	0.936467397
PAK2 (Phospho-Ser192)	0.744913288	1.042969912	0.700615296
PAK2 (Phospho-Ser20)	0.837813047	0.905720695	1.039586934
PAK3 (Phospho-Ser154)	0.733200554	1.31944233	0.837056694
PAK4/PAK5/PAK6 (Ab-474)	4.612869906	0.919798026	4.942115134
PI3-kinase p85-alpha (Phospho-Tyr607)	1.693119797	2.029396911	1.978413752
PI3-kinase p85-subunit alpha/gamma (Phospho-Tyr467/199)	1.839260318	1.808839608	1.960828143
PKC alpha (Phospho-Tyr657)	1.209459152	0.893405033	1.300038528
PKC alpha/beta II (Phospho-Thr638)	0.889897339	0.954054295	0.700405406
PKC beta/PKCB (Phospho-Ser661)	0.873420822	1.194608994	0.858150778
PKC delta (Phospho-Ser645)	1.013453322	1.087438512	1.093763769
PKC delta (Phospho-Thr505)	1.163533934	1.10911549	0.98057754
PKC epsilon (Phospho-Ser729)	0.840096885	0.802475046	0.942721001
PKC theta (Phospho-Ser676)	0.690810743	0.755507852	0.564922354
PKC theta (Phospho-Thr538)	0.884019638	0.745745063	1.080936772
PKC zeta (Phospho-Thr410)	1.45433595	1.146406475	1.315959719
PKC zeta (Phospho-Thr560)	0.954133637	0.871373125	0.875524324
PP2A-a (Phospho-Tyr307)	1.536101787	1.184362941	1.276083888
Rac1/cdc42 (Phospho-Ser71)	1.350777235	1.220270261	1.278789966
RAS(p21 H and K) (Inter)	4.110604574	1.026068954	4.917477218
RASE (Inter)	4.436054506	0.934526651	4.220950481
RASF4 (Inter)	4.479216717	1.024552914	3.155660496
Ras-GRF1 (Phospho-Ser916)	0.690823892	0.707703944	0.633934676
Rho/Rac guanine nucleotide exchange factor 2 (P-Ser885)	1.2373093	1.733821019	1.228935103
RhoA (Ab-188)	5.776367233	1.052621426	5.523850156
S6 Ribosomal Protein (Phospho-Ser235)	1.067921413	1.166579364	1.218080476
S6K (Inter)	4.594226461	1.026468453	3.398369235
S6K-alpha 6 (Inter)	4.001553725	0.947254126	2.561237052
SAPK/JNK (Phospho-Thr183)	1.040166043	1.02682128	1.180933991
SAPK/JNK (Phospho-Tyr185)	1.207609654	1.158984375	1.370725643
SEK1/MKK4 (Phospho-Ser80)	1.477017413	0.42066294	1.163517253
SEK1/MKK4 (Phospho-Thr261)	1.474118436	0.428207448	1.157715216
Shc (Phospho-Tyr349)	1.265148708	1.189230496	1.420817389
Shc (Phospho-Tyr427)	1.211281438	0.968122813	1.375704356
Smad4 (Inter)	4.043549773	0.997253483	3.028670476
SP1 (Phospho-Thr739)	1.104641629	1.077601639	1.012966324
TAK1 (Phospho-Thr184)	1.362524462	1.256140865	1.833035339
TGF alpha (inter)	2.292945365	2.363234255	1.876366319
TGF beta receptor II (inter)	8.136442304	0.808232283	6.032782418
TGF beta1 (inter)	4.714765745	1.100785087	4.698498539
TGF beta2 (inter)	7.069229694	0.972243919	15.30892946
TGF beta3 (inter)	4.391364975	1.075187635	3.669686859
TGFBR1 (Ab-165)	2.759087366	0.667630964	2.617034205
TGFBR2 (Ab-250)	2.834546943	0.650381798	3.389708845
